# A Simple Test for the Detection of Albumin in the Urine1Read at the meeting of the New York State Veterinary Medical Society.

**Published:** 1899-07

**Authors:** Pierre A. Fish

**Affiliations:** Ithaca, New York


### A SIMPLE TEST FOR THE DETECTION OF ALBUMIN IN URINE.[1]

By Pierre A. Fish, D.Sc., D.V.S.,
ITHACA, NEW YORK.

Among those agents which have the property of coagulating or precipitating albumin, alcohol holds a prominent position. Its use in the detection of albumin in urine does not seem to have been practised very extensively, if at all.

The following tests with alcohol have been tried upon urine containing albumin, and upon control-solutions of albumin in distilled water. In one set of experiments some dry albumin from blood was dissolved in the water; in another set, some blood-serum was added to the water. (The proportion of serum-albumin to the serum is approximately 1 to 10.)

If the alcohol be added to normal omnivorous urine a precipitation of the phosphates will occur; the addition of a few drops of nitric acid will cause their disappearance. If the urine contain albumin and the nitric acid be added after the alcohol, the precipitated albumin will remain, the phosphate having gone into solution.

[1] Read at the meeting of the New York State Veterinary Medical Society.

In order to determine the delicacy of the tests, the following solutions were prepared and tested: Solutions of dry albumin from the blood were prepared as follows: 1 part of albumin to 1000 parts of water, 1 to 2000, 1 to 4000, 1 to 8000, 1 to 16,000, 1 to 32,000, 1 to 64,000. The addition of the alcohol alone caused precipitates up to 1 to 32,000, although, in the latter, the precipitate was only just perceptible; after the addition of nitric acid, the precipitates were less dense and had disappeared in the 1 to 32,000 tube, due probably to the formation of acid-albumin and going into solution. After heating, the appearances remained about the same. The tubes were left in place over night, and when next examined, the precipitates were more distinct, and a precipitate had appeared in the 1 to 32,000 tube, and even in the 1 to 64,000 tube small flecks of albumin could be detected in suspension.

The experiment was repeated in a little different form, merely substituting some blood-serum for the dry albumin. Ten cubic centimeters of the serum were added to 1 litre of distilled water, which gave, approximately, 1 part of serum-albumin to 1000 of water. The proportions were arranged as in the preceding set of experiments, and the same results were obtained.

Two other sets of experiments were performed to compare the relative delicacy of nitric acid and alcohol. The alcohol gave a precipitate in the 1 to 32,000 tube and the nitric acid a precipitate in the 1 to 16,000 tube.

The effect of adding alcohol after removing the phosphates was also tried; some ammonia-water was added to omnivorous urine to precipitate the earthy phosphates (calcium and magnesium). The addition of the alcohol to the urine, after the removal of the earthy phosphates by precipitation and filtration, gave but a small amount of precipitate as compared with the original urine. This precipitate was evidently composed of the alkaline phosphates (sodium and potassium), which are insoluble in alcohol. After the removal of both *earthy* and *alkaline* phosphates, no precipitate occurred when alcohol was added.

The sulphates and chlorides of potassium and sodium are converted, by the ammonia-water used to precipitate the earthy phosphates, into the corresponding salts of ammonium, and these are soluble in the alcohol diluted by the urine.

The test, in brief, is as follows: Add to the suspected urine, one-half the volume of strong alcohol. The fluid will become cloudy and a precipitate may form; add a little nitric acid and heat. If the precipitate disappears it is composed of the phosphates; if it remains, it is albumin.

It would seem from the experiments performed, that neither alcohol nor nitric acid alone can be depended upon for strictly accurate results; but that a combination of the two as proposed in the above test, is a distinct advantage,—the alcohol to prevent or lessen the tendency for the formation of acid-albumin, and to obtain a more delicate test than can be obtained from nitric acid alone; the nitric acid to keep the phosphates in solution, and, also, by acting upon the chlorides and sulphates, to put them in a more soluble form in the urine-diluted alcohol.

Mucin is also coagulated by alcohol, and urates may be deposited after the urine has stood for a time. These should not be confused with albumin, since they disappear upon the application of heat, while albumin is coagulated.

---

Veterinary practice all over the land was much better this spring and summer than it has been for a number of years.

Belfast, Ireland, has had so serious an outbreak of glanders among horses that stringent police measures as to all public watering-fountains, stands, hitching-posts, etc., have had to be inaugurated.

London, England, will witness shortly a dog-show of canines owned exclusively by physicians, the proceeds for the benefit of a charitable institution.

The members of the medical profession in certain States are up in arms against the encroachment on their rights by a new species of medical charlatans, styling themselves "Osteopaths." What with "Christian Science Healing," "Spiritualism," the patent-medicine fakir, and the Metropolitan free dispensaries and other medical fads, the pathway of the medical practitioner to-day is far from being strewn with roses.

From Antwerp, Belgium, comes the announcement that a serum for the cure and eradication of cancer has been discovered and successfully employed.

